# Ferroelectric Diode Effect with Temperature Stability of Double Perovskite Bi_2_NiMnO_6_ Thin Films

**DOI:** 10.3390/nano9121783

**Published:** 2019-12-15

**Authors:** Wen-Min Zhong, Qiu-Xiang Liu, Xin-Gui Tang, Yan-Ping Jiang, Wen-Hua Li, Wan-Peng Li, Tie-Dong Cheng

**Affiliations:** School of Physics Optoelectric Engineering, Guangdong University of Technology, Guangzhou Higher Education Mega Center, Guangzhou 510006, China; zhongwen_min@163.com (W.-M.Z.); liuqx@gdut.edu.cn (Q.-X.L.); ypjiang@gdut.edu.cn (Y.-P.J.); liwenhuat@gdut.edu.cn (W.-H.L.); liwanpeng361@163.com (W.-P.L.); chengtiedong@126.com (T.-D.C.)

**Keywords:** Bi_2_NiMnO_6_, thin films, diode effect, oxygen defect, conduction mechanism

## Abstract

Double perovskite Bi_2_NiMnO_6_ (BNMO) thin films grown on p-Si (100) substrates with LaNiO_3_ (LNO) buffer layers were fabricated using chemical solution deposition. The crystal structure, surface topography, surface chemical state, ferroelectric, and current-voltage characteristics of BNMO thin films were investigated. The results show that the nanocrystalline BNMO thin films on p-Si substrates without and with LNO buffer layer are monoclinic phase, which have antiferroelectric-like properties. The composition and chemical state of BNMO thin films were characterized by X-ray photoelectron spectroscopy. In the whole electrical property testing process, when the BNMO/p-Si heterojunction changed into a BNMO/LNO/p-Si heterojunction, the diode behavior of a single diode changing into two tail to tail diodes was observed. The conduction mechanism and temperature stability were also discussed.

## 1. Introduction

In the past few decades, electronic devices prepared using a semiconductor have become an important research project in the field of materials science [1,2,3,4]. These devices have garnered attention for their practical applications, such as magnetoresistance, photodetectors, p-n diodes and thin film transistors [5,6]. Bi_2_NiMnO_6_ has been widely studied as a multiferroic material. The ferromagnetic and ferroelectric Bi_2_NiMnO_6_ was successfully prepared at 6 GPa as reported by Azuma et al. [7]. Low temperature (about 100 K) ferroelectric properties in pulsed laser-deposition drive Bi_2_NiMnO_6_ thin films on (001)-oriented SrTiO_3_ single crystal substrates were reported by Sakai et al. [8]. The phase transition temperature of epitaxial Bi_2_NiMnO_6_ thin films affected by single crystal substrates was studied using Raman spectroscopy [9]. The magnetodielectric effect was obtained in single-phase and epitaxial thin film of multiferroic Bi_2_NiMnO_6_, as reported by Padhan et al. and Rathi et al. [10,11,12]. The ferroelectric behavior and magnetic exchange interaction effect of Bi_2_NiMnO_6_ with the electric polarization 19.01 μC/cm^2^ was reported by Zhao et al. [13]. Theoretical and experimental results confirm that Bi_2_NiMnO_6_ thin films are multiferroic materials [8,14,15,16,17,18]. The ferroelectric and current leakage characteristics of La-doped Bi_2_NiMnO_6_ and Bi_2_NiMnO_6_ thin film was reported by Li et al. [19].

However, the ferroelectric diode effect and temperature stability of Bi_2_NiMnO_6_ thin film has never been reported. Therefore, in this work, a thin film of Bi_2_NiMnO_6_ was growth on p-Si and LaNiO_3_/p-Si substrates using chemical solution deposition technology, the ferroelectric diode effect and temperature stability of Bi_2_NiMnO_6_ thin film was first investigated, as was the conduction mechanism.

## 2. Materials and Methods

The Bi_2_NiMnO_6_ (abbreviated as BNMO) precursor was prepared by dissolving nitrogen salt, bismuth, manganese acetate, and nickel acetate in a ratio of 2.2:1:1 in ethylene glycol solution [16]. The excess of 10% bismuth was to prevent evaporation of the film during the drying and annealing process. Three milliliters of acetic acid was added to the solution to prevent precipitation. The precursor BNMO concentration is 0.2 M. The LaNiO_3_ (LNO) precursor was prepared by dissolving nickel acetate and lanthanum nitrate in a ratio of 1:1 in ethylene glycol solution and the resulting concentration was 0.3 M. The 5 mL acetone acetate stabilizer was also added to the precursor. The precursors were then aged for 3 days. The wet LNO/p-Si thin film was synthesized by a spin-coating process at a rate of 2500 rpm for 15 s. The LNO/p-Si substrate was made using a drying process at 573 K for 5 min and an annealing process at 973 k for 30 min. The BNMO/p-Si and BNMO/LNO/p-Si heterojunctions are prepared using the spin coating process at 3000 rpm for 15 s, the dry process at 573 K for 5 min and annealing process at 973 K for 10 min by rapid thermal annealing (RTA) in air atmosphere. A gold electrode with a diameter of 0.3 mm was plated on the surface of the film sample by a small high vacuum coater to form a film capacitor structure.

The crystal structure analysis was measured using XRD (Bruker D8 Advance, AXS, Germany) and the chemical states were examined using X-ray photoelectron spectroscopy (XPS, Escalab 250Xi, Sussex, UK). The surface topography and elemental analysis were performed with the field emission scanning electron microscope (FE-SEM, SU8220, Hitachi, Japan). The ferroelectric properties of the BNMO thin films were measured with a ferroelectric test system (Radiant Technologies Precision Workstation, Albuquerque, NM, USA). The current-voltage characteristic was measured with a Keithley 2400 system.

## 3. Results and Discussion

**Figure 1:**
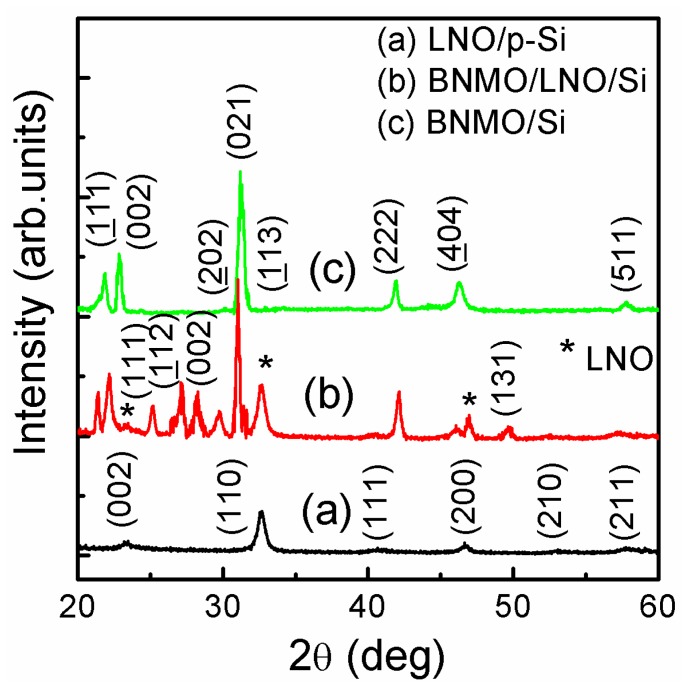
The XRD patterns of LNO, BNMO/LNO and BNMO thin films on p-Si substrates.

The XRD patterns are shown in Figure 1. From Figure 1, there are six peaks at (100), (110), (111), (200), (210) and (211), the crystal structure of LNO grown on p-Si can be judged by PDF card of 33-0710 as a cubic phase. The crystal structure of BNMO film grown on p-Si substrate without and with LNO can be determined by diffraction angles of 23.62° and 31.18°. It has the monoclinic structure reported by Azuma et al. [7]. The unit cell of BNMO was considered to be similar to BiMnO_3_. The three possible transition metal sites of M1, M2, and M3 were filled with Bi^3+^, Ni^2+^ and Mn^4+^ cations and the Mn^4+^–O^2−^–Ni^2+^ chemical links and Bi^3+^–O^2−^ links were the main chain segment. The diffraction angle of 27.58°and 29.22° matches the results of Li et al. [19], and it is a monoclinic structure with *C*2 space group [20]. In theory, The NiO_6_ and MnO_6_ octahedral of BNMO are isotropic and do not cause distortion.

**Figure 2:**
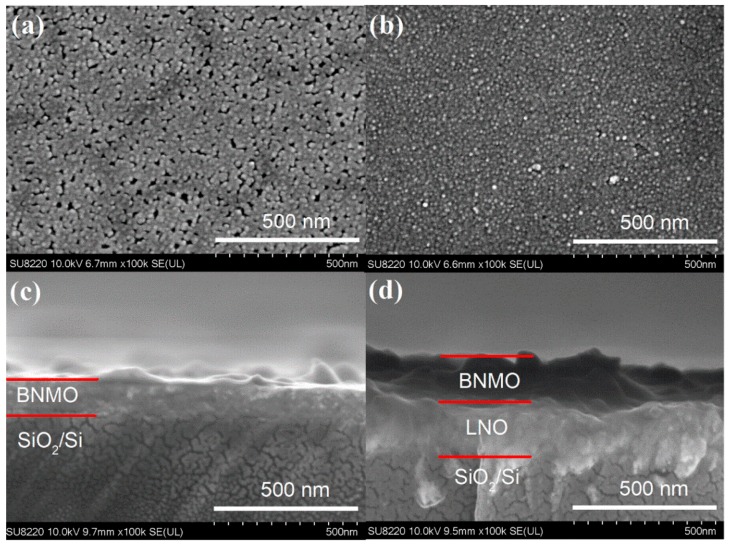
The surface topography and cross-section images: (**a**,**c**) for BNMO/p-Si, (**b**,**d**) for BNMO/LNO/p-Si.

Figure 2 show the surface and cross-section topographies of BNMO thin film and BNMO/LNO thin film grown p-Si substrates, respectively. From Figure 2a,b it can be clearly observed from the surface topography that the grain size of BNMO/p-Si thin film is nearly 15 nm, but the BNMO/LNO/p-Si thin film is nearly 10 nm. The BNMO thin film was formed by the Mn^4+^ cations and Ni^2+^ cations, and the thin films was annealed at air atmosphere for only 10 min. Therefore, the growth of crystal grains is relatively difficult, resulting in a small grain size. Observed from the cross-section images, it can be obtained that the thickness of the BNMO layer growth on the p-Si substrata is nearly 100 nm (see Figure 2c), the BNMO layer on LNO/p-Si substrate is 140 nm (see Figure 2d). The different film thickness of the BNMO layers may be caused by the different adhesion of LNO and Si to the solution and the first layer, respectively.

**Figure 3:**
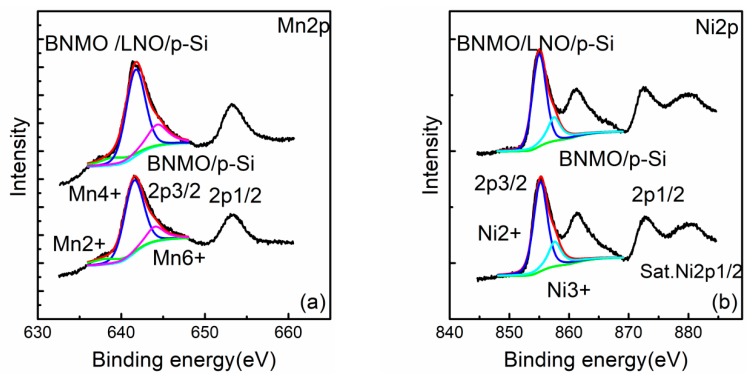
The fitted narrow-scan spectra for (**a**) Mn 2p and (**b**) Ni 2p.

The XPS spectra of Ni 2p and Mn 2p are shown in Figure 3. The binding energy of Mn 2p_3/2_ and Mn 2p_1/2_ of the BNMO/p-Si heterojunctions was 641.35 eV and 653.2 eV [21]. The binding energy of Mn 2p_3/2_ and Mn 2p_1/2_ of the BNMO/LNO heterojunction was 641.6 eV and 653.45 eV. The binding energy of Ni 2p_3/2_ and Ni 2p_1/2_ of the BNMO/p-Si heterojunction was 872.45 eV and 861.3 eV [22]. The binding energy of Ni 2p_3/2_ and Ni 2p_1/2_ of the BNMO/LNO/p-Si heterojunction was 855.1 eV and 872.55 eV.

The XPS spectrum is subject to peak processing. The binding energy of 638.85 eV, 641.65 eV, and 644.25 eV of the BNMO/p-Si heterojunction and 638.25 eV, 641.5 eV, and 643.95 eV of the BNMO/LNO/p-Si heterojunction indicates the Mn^2+^, Mn^4+^ and Mn^6+^ cation. The binding energy of 855 eV and 857.65 eV of the BNMO/p-Si heterojunction and 855 eV and 857.2 eV of the BNMO/LNO/p-Si heterojunction shows the Ni^2+^ and Ni^3+^ cation. The ion ratio of Mn^2+^:Mn^4+^:Mn^6+^ on BNMO/p-Si thin film is 0.15:1:0.3 and for BNMO/LNO/p-Si thin film is 0.16:1:0.27. The ratio of Ni^2+^:Ni^3+^ on the BNMO/p-Si heterojunction and on the BNMO/LNO heterojunction is 1:0.22 and 1:0.24, respectively. By analyzing the XPS spectrum, a variety of Ni, Mn ions are found in the BNMO heterojunction device. The cubic crystal structure of NiO and BiMnO_3_ octahedral crystallites interferes with the formation of pure phase monoclinic crystals and causes crystal defects.

**Figure 4:**
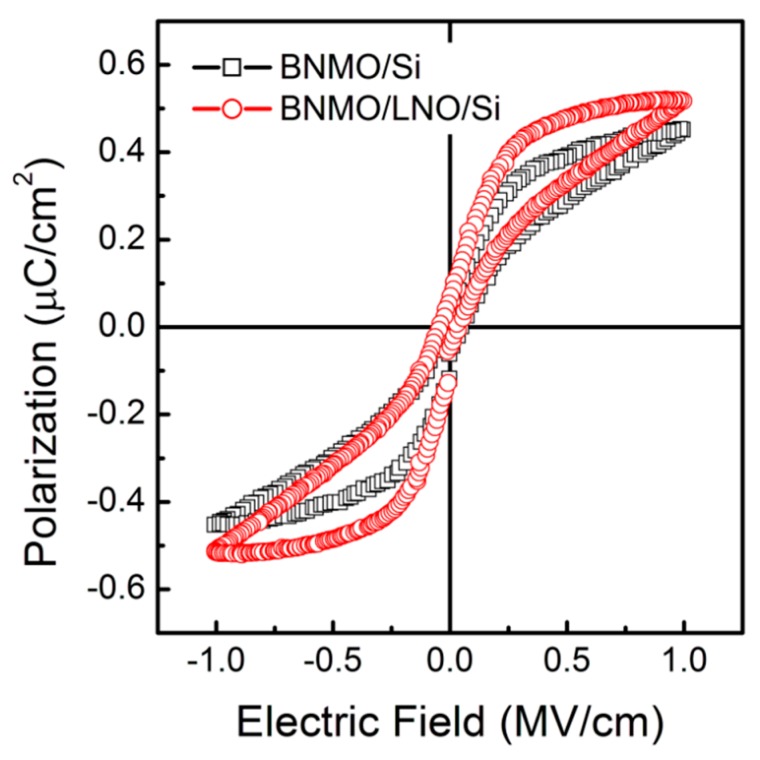
The typical hysteresis loops of the BNMO/Si and BNMO/LNO/Si thin films measured at 500 Hz.

The room temperature polarization-electric field (*P-E*) properties are shown in Figure 4. The results show that the two films have antiferroelectric-like properties. The saturated polarization (2*P_s_*), remnant polarization (2*P_r_*), and coercive field (*E_c_*) of BNMO/LNO/Si thin film were 0.875 µC/cm^2^, 0.150 µC/cm^2^, and 40.0 kV/cm, and 1.03 µC/cm^2^, 0.202 µC/cm^2^, and 38.4 kV/cm, respectively for BNMO/Si and BNMO/LNO/Si thin films at 500 Hz. The ferroelectric polarization was enhanced by using LNO as a buffer layer. The room temperature ferroelectric polarization phenomenon could be due to the incompletely symmetric monoclinic structure preventing the ferroelectric domain from flipping. It is a pinning effect caused by the interaction of defect dipoles in the BNMO layer. The BNMO growth on the LNO/p-Si substrate is a nanocrystalline state, resulting in more lattice defects, preventing the deformation of the crystal [7].

**Figure 5:**
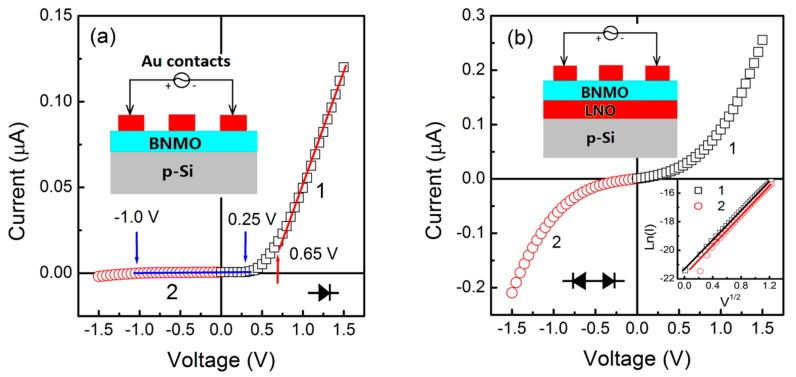
I-V characteristics of BNMO thin films without (**a**) and with (**b**) a LNO buffer layer on p-Si substrates. Insets show the schematics of the diode cell used for measurement.

The current-voltage characteristics of BNMO/Si and BNMO/LNO/Si thin films are shown in Figure 5. From Figure 5a, we know that from 0 to −1.0 V and from 0 to 0.25 V, the current hardly changed with the increase of absolute voltage value, when the voltage increases from −1.0 to −1.5 V, the current increased, and when the voltage increased from 0.65 to 1.5 V, the current increased rapidly. The results show typical diode characteristics.

The BNMO/p-Si heterojunction exhibited rectification effect behavior of p-n junction. It is a forward-conducting heterojunction device with an ON/OFF ratio (*R* = *I*_on_/*I*_off_) of 65. The n-type semiconductor properties of BNMO have not been reported. It can be inferred from the results, and the process of converting Mn^2+^ ions into Mn^4+^ ions can release electrons.

As the bottom electrodes of the LNO grew on the p-Si substrate, the rectification effect of the forward conduction was forcibly converted into a reverse conduction rectification effect, and the ON/OFF ratio is increased to 1.4. The result shows two tail to tail diodes. The cubic phase of LNO acted as an n-type semiconductor to release electrons, while the nano-crystallinity of the BNMO layer defects acted as a hole-absorbing electron [23,24,25].The Schottky emission mechanism is determined by the linear relationship of Ln(*I*) versus V^1/2^ [26,27]. If the relationship is linear, this is due to the thermionic emission by holes, vacancies and defects [28,29,30,31,32], respectively. The restricted behavior of the interface and the hole trapping behavior are considered to be a case of the Schottky emission mechanism, as expected with linear relationship of Ln(*I*) versus V^1/2^ for the BNMO/LNO/p-Si heterojunction (see the inset of Figure 5b). Therefore, the large ON/OFF ratio of the BNMO/LNO/p-Si heterojunction is caused by the hole of the BNMO layer being filled.

**Figure 6:**
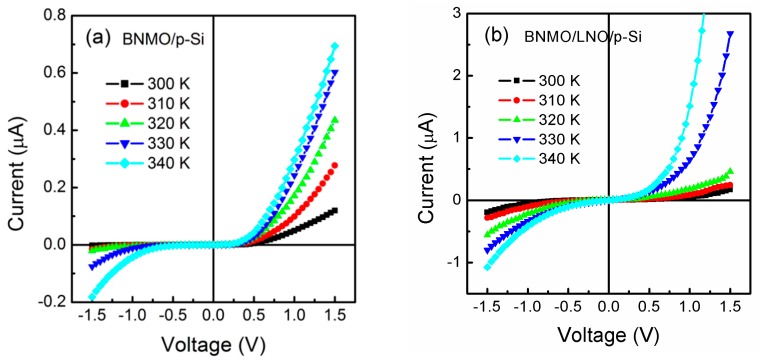
The I-V characteristics of BNMO thin films (**a**) without and (**b**) with a LNO buffer layer on p-Si substrates measured at different temperatures.

The voltage-current characteristics measured at different temperatures were shown in Figure 6. The conductivity of the BNMO/p-Si heterojunction and the BNMO/LNO/p-Si heterojunction was increased due to increased test temperature. The ON/OFF ratio of the BNMO/p-Si heterojunction is decreased from 65 to 3.8, and the ON/OFF ratio of the BNMO/LNO/p-Si heterojunction is increased from 1.4 to 13.4. The result can be interpreted with the thermion emission effect of Schottky diodes [33,34,35,36]. The electrons are excited by the lattice defects caused by the heat. Therefore, the conductivity of the film increases. At the same time, the space charge accumulated at the interface of the heterojunction is also subject to thermal radiation. The BNMO/p-Si heterojunction is excited by thermal radiation, causing the electrons of the defect to be excited, forming a space charge region at the interface of the heterojunction, and finally the rectification effect is improved. The BNMO/LNO/p-Si heterojunction is thermally radiated, and the electron trapping ability of the hole is weakened, resulting in the rectification effect being weakened.

## 4. Conclusions

In conclusion, the BNMO thin films on Si and LNO/Si substrates show a monoclinic phase with *C2* space group and the porosity of BNMO thin film with LNO layer is smaller than without, resulting in a larger ferroelectric polarization. The conduction mechanism of the BNMO/Si and BNMO/LNO/Si heterojunctions were dominated by Ohmic conduction and Schottky emission mechanisms, respectively. In the case of temperature rise, the rectification effect of BNMO/Si will decrease due to the energy of the hole trapping electrons being weakened and the rectification effect of the BNMO/LNO/p-Si heterojunction will increase due to the charge accumulation, respectively. XPS testing shows that BNMO synthesized under normal pressure is a material with the coexistence of different valence cations.
